# Imbalance in the Blood Concentrations of Selected Steroids in Pre-pubertal Gilts Depending on the Time of Exposure to Low Doses of Zearalenone

**DOI:** 10.3390/toxins11100561

**Published:** 2019-09-25

**Authors:** Anna Rykaczewska, Magdalena Gajęcka, Ewa Onyszek, Katarzyna Cieplińska, Michał Dąbrowski, Sylwia Lisieska-Żołnierczyk, Maria Bulińska, Andrzej Babuchowski, Maciej T. Gajęcki, Łukasz Zielonka

**Affiliations:** 1 Department of Veterinary Prevention and Feed Hygiene, Faculty of Veterinary Medicine, University of Warmia and Mazury in Olsztyn, Oczapowskiego 13/29, 10-718 Olsztyn, Poland; anna.rykaczewska@uwm.edu.pl (A.R.); michal.dabrowski@uwm.edu.pl (M.D.); gajecki@uwm.edu.pl (M.T.G.); lukaszz@uwm.edu.pl (Ł.Z.); 2 Dairy Industry Innovation Institute Ltd., 11-700 Mrągowo, Poland; ewa.onyszek@iipm.pl (E.O.); andrzej.babuchowski@iipm.pl (A.B.); 3 Microbiology Laboratory, Non-Public Health Care Centre, ul. Limanowskiego 31A, 10-342 Olsztyn, Poland; kasiacieplinska@gmail.com; 4 Independent Public Health Care Centre of the Ministry of the Interior and Administration, and the Warmia and Mazury Oncology Centre in Olsztyn, Wojska Polskiego 37, 10-228 Olsztyn, Poland; lisieska@wp.pl; 5 Department of Discrete Mathematics and Theoretical Computer Science, Faculty of Mathematics and Computer Science, University of Warmia and Mazury in Olsztyn, Słoneczna 34, 10-710 Olsztyn, Poland; bulma@uwm.edu.pl

**Keywords:** zearalenone, low doses, steroid hormones, biotransformation, pre-pubertal gilts

## Abstract

Zearalenone (ZEN) is a mycotoxin that not only binds to estrogen receptors, but also interacts with steroidogenic enzymes and acts as an endocrine disruptor. The aim of this study was to verify the hypothesis that low doses, minimal anticipated biological effect level (MABEL), no-observed-adverse-effect level (NOAEL) and lowest-adverse-effect level (LOAEL), of ZEN administered orally for 42 days can induce changes in the peripheral blood concentrations of selected steroid hormones (estradiol, progesterone and testosterone) in pre-pubertal gilts. The experiment was performed on 60 clinically healthy gilts with average BW of 14.5 ± 2 kg, divided into three experimental groups and a control group. Group ZEN5 animals were orally administered ZEN at 5 μg ZEN/kg BW, group ZEN10 — at 10 μg ZEN/kg BW, group ZEN15 — at 15 μg ZEN/kg BW, whereas group C received a placebo. Five gilts from every group were euthanized on analytical dates 1, 2 and 3 (days 7, 14 and 42 of the experiment). Qualitative and quantitative changes in the biotransformation of low ZEN doses were observed. These processes were least pronounced in group ZEN5 (MABEL dose) where ZEN metabolites were not detected on the first analytical date, and where β-ZEL was the predominant metabolite on successive dates. The above was accompanied by an increase in the concentration of estradiol (E_2_) which, together with “free ZEN”, probably suppressed progesterone (P_4_) and testosterone (T) levels.

## 1. Introduction

Zearalenone (ZEN) is a macrocyclic lactone of β-resorcylic acid with clear estrogen activity. This non-steroidal estrogenic mycotoxin is produced by several species of the genus *Fusarium*. Zearalenone is metabolized to numerous derivatives by microorganisms, plants, animals and humans. Previous research into the metabolism of ZEN revealed the presence of reducing metabolites, in particular α-zearalenol (α-ZEL) and its stereoisomer, β-zearalenol (β-ZEL) [1]. When these catechol metabolites are synthesized, the activity of ZEN resembles that of endogenous estrogens, such as estradiol (E_2_). As a result, ZEN can affect reproduction in pre-pubertal gilts, the expression of hydroxysteroid dehydrogenases (HSDs) and the synthesis and secretion of sex hormones, including E_2_, progesterone (P_4_) and testosterone (T) [2,3]. These unverified facts have become the reason for the studies. This was accompanied by other doubts of tape – that this mycotoxin should not be tested using the minimal anticipated biological effect level (MABEL) dose, the no-observed-adverse-effect level (NOAEL) dose [4] and/or the lowest-adverse-effect level (LOAEL) dose [5]. Mammals have adapted to prolonged exposure to low monotonic doses of ZEN [6], or have even learned to exploit this mycotoxin in their physiological processes [7,8,9].

At the beginning of this decade, the classical dose-response paradigm was undermined by the “low dose hypothesis”, in particular, with regard to hormonally active substances [6] which act as endocrine disruptors (EDs) and/or disrupt paracrine and endocrine signaling [1]. These processes can be observed during exposure to low doses of undesirable substances which are present in food and feed [10,11] and which induce differential responses in macroorganisms (hormesis [12]). These dose-response interactions remain relatively unexplored, and the risks (clinical symptoms or laboratory results) associated with high doses cannot be clearly extrapolated to low doses [13,14] that deliver counterintuitive effects.

From the point of view of biomedical practice [15], the MABEL concept is garnering increasing interest because the clinical picture is influenced by numerous endogenous factors. The clinical picture reflects not only disruptions in the steroid hormone balance or the quantity and quality of exogenous hormone-like substances [16], but also other somatic responses, including reproductive behavior [1], immune responses [17] and changes in the metabolic profile of peripheral blood [9]. A thorough understanding of the relevant mechanisms of action and the final effects supports sound decision-making [14]. Substances that disrupt hormonal homeostasis have undermined long-standing paradigms in toxicology, in particular the “dose makes the poison” concept [18]. Low doses of ZEN and its metabolites (ZELs) induce specific changes [7,8,9] that are not encountered during exposure to high doses. Research into natural hormones and EDs has demonstrated that low doses produce ambiguous responses (pro-inflammatory, anti-inflammatory, increase or decrease in proliferative activity [19]). The above also applies to ZEN [1,20,21].

In view of the above, the aim of this study was to validate the hypothesis that MABEL, NOAEL and LOAEL doses of ZEN administered orally for 42 days can induce changes in peripheral blood concentrations of selected steroid hormones (estradiol, progesterone and testosterone) in pre-pubertal gilts.

## 2. Results

### 2.1. Experimental Feed

The analyzed feed did not contain mycotoxins, or its mycotoxin content was below the sensitivity of the method (VBS). The concentrations of modified and masked mycotoxins were not analyzed.

### 2.2. Clinical Observations

Clinical signs of ZEN mycotoxicosis were not observed throughout the experiment. However, changes in specific tissues or cells were frequently observed in analyses of the serum biochemical profile, caecal water genotoxicity and intestinal microbiome parameters in samples collected from the same animals and in those animals’ growth performance. The results of these analyses were published in a different paper [7,8,9].

### 2.3. The Effect of Various Doses of Zearalenone on Hormone Secretion

#### 2.3.1. The Effect of Estradiol

**Figure 1:**
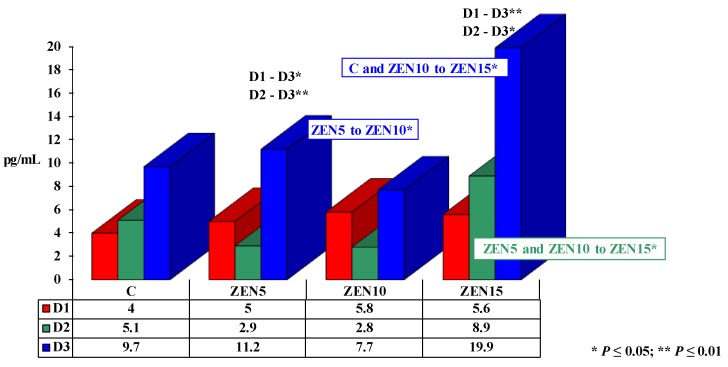
The effect of zearalenone (ZEN) on estradiol (E_2_) concentration in the blood of pre-pubertal gilts: arithmetic means ($\bar{x}$) of five samples collected on each analytical date (D1, D2 and D3) in every group (control (C), ZEN5, ZEN10 and ZEN15). Statistically significant differences were determined at *P ≤ 0.05 and **P ≤ 0.01.

The concentration of E_2_ in the blood of pre-pubertal gilts (see Figure 1) ranged from 2.9 to 19.9 pg/mL. Significant differences (see Figure 1) were observed between analytical dates in groups and between groups on different dates. In the control group (C), E_2_ levels increased by 5.7 pg/mL during the experiment. Estrogen concentration also increased in all experimental groups during the entire period of exposure (by 6.2 pg/mL in group ZEN5; by 1.9 pg/mL in group ZEN10 – a value lower than in group C; by 14.3 pg/mL in group ZEN15), but the reference level on D1 was higher in the experimental groups than in group C. On D2, the concentration of E_2_ decreased in groups ZEN5 and ZEN10, relative to D1.

On different analytical dates (see Figure 1), the concentration of E_2_ increased in the experimental groups relative to group C (from 1.8 pg/mL on D1, through 3.8 pg/mL on D2 to 10.2 pg/mL on D3), proportionally to the applied mycotoxin dose. Estrogen levels decreased in groups ZEN5 (difference of 2.2 pg/mL) and ZEN10 (difference of 2.3 pg/mL) on date D2, and in group ZEN10 (difference of 2.0 pg/mL) on date D3, relative to group C.

A comparison of the mean ($\bar{x}$) concentrations of E_2_ in groups during the entire experiment revealed the lowest values in group ZEN10 (5.4 pg/mL). In the remaining experimental groups, E_2_ levels were higher than in group C, proportionally to the administered dose of ZEN (from 6.2 pg/mL in group C and 6.4 pg/mL in group ZEN5 to 11.5 pg/mL in group ZEN15).

#### 2.3.2. The Effect of Progesterone

**Figure 2:**
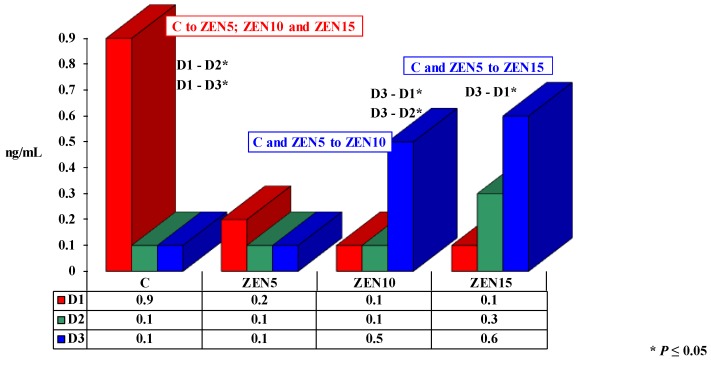
The effect of ZEN on progesterone (P_4_) concentration in the blood of pre-pubertal gilts: arithmetic means ($\bar{x}$) of five samples collected on each analytical date (D1, D2 and D3) in every group (C, ZEN5, ZEN10 and ZEN15). Statistically significant differences were determined at *P ≤ 0.05.

Progesterone concentrations ranged from 0.1 to 0.9 ng/mL during the experiment (Figure 2).

Significant differences in progesterone concentrations are presented in Figure 2. In group C, P_4_ levels decreased by 0.7 ng/mL and 0.8 ng/mL between D1 versus D2 and D3, respectively. In group ZEN5, P_4_ concentration remained stable at approximately 0.1 ng/mL. In the remaining experimental groups, a steady increase in P_4_ levels was observed over time (to 0.5 ng/mL in group ZEN10; to 0.6 ng/mL in group ZEN15).

On D1, progesterone concentrations were much lower in the experimental groups than in group C (by 0.7; 0.8 and 0.8 ng/mL, respectively). On D2, P_4_ values were similar in group C and groups ZEN5 and ZEN10 (0.1 ng/mL lower in both groups than in group C). In group ZEN15, the concentration of P_4_ increased to 0.3 ng/mL, and it was 0.2 ng/mL higher than in group C. On D3, P_4_ levels were low and similar in groups C and ZEN5 at around 0.1 ng/mL. In groups ZEN10 and ZEN15, the noted values were much higher than in group C (by 0.5 and 0.6 ng/mL, respectively).

The mean concentration of P_4_ in each group was very low on each analytical date. The mean values of P_4_ in all experimental groups were lower than in group C (0.3 ng/mL in group C; 0.1 ng/mL in group ZEN5; 0.2 ng/mL in group ZEN10; 0.3 ng/mL in group ZEN15), proportionally to the administered dose of ZEN.

#### 2.3.3. The Effect of Testosterone

**Figure 3:**
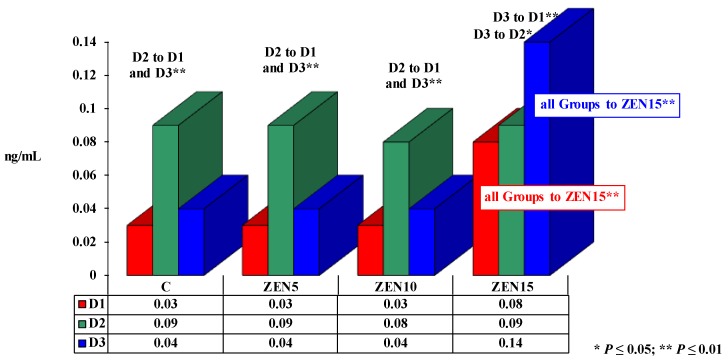
The effect of ZEN on testosterone (T) concentration in the blood of pre-pubertal gilts: arithmetic means ($\bar{x}$) of five samples collected on each analytical date (D1, D2 and D3) in every group (C, ZEN5, ZEN10 and ZEN15). Statistically significant differences were determined at *P ≤ 0.05 and **P ≤ 0.01.

Significant differences in testosterone concentrations are presented in Figure 3. Testosterone levels ranged from 0.03 to 0.14 ng/mL during the experiment.

In all groups, testosterone levels were lowest at the beginning of the experiment, and they increased over time of exposure to ZEN. Similar changes were observed in groups C, ZEN5 and ZEN10, but a rapid increase in T levels was noted on D2. Testosterone concentrations increased gradually over time from 0.08 ng/mL on D1, to 0.09 ng/mL on D2 and 0.14 ng/mL on D3 only in group ZEN15.

On D1 and D3, T levels were highly similar at 0.03–0.04 ng/mL, excluding in group ZEN15 where they were very high at 0.08 ng/mL on D1 and 0.14 ng/mL on D3. On D2, T concentrations ranged from 0.08 to 0.09 ng/mL in all groups, and the highest value was noted in group C.

During the experiment, the mean concentration of T was highest in group ZEN15 (0.102 ng/mL). Testosterone levels were lower in the remaining groups (from 0.05 ng/mL in group C and 0.05 ng/mL in group ZEN5 to 0.05 ng/mL in group ZEN10).

#### 2.3.4. Pearson’s Correlation Coefficient (r)

The values of *r* were calculated based on the concentrations of steroid hormones (estradiol – E_2_, progesterone – P_4_ and testosterone – T) in the blood of pre-pubertal gilts on different analytical dates and in different groups. The coefficients of correlations between the concentrations of E_2_ and P_4,_ E_2_ and T, and P_4_ and T are presented in Table 1, Table 2 and Table 3, respectively [22]. A positive correlation (*r* > 0) was determined when an increase in the concentration of one hormone led to a rise in the concentration of another hormone. The strength of positive correlations was determined on the following scale: *r* < 0.2 — no correlation, *r* = 0.2 to 0.4 — weak correlation, *r* = 0.4 to 0.7 — moderate correlation, *r* = 0.7 to 0.9 — relatively strong correlation, and *r* > 0.9 – very strong correlation. The correlation coefficient was determined at 0.0 only when the concentration of P_4_ on a given analytical date was below the sensitivity of the method. A negative correlation (*r* < 0) was determined when an increase in the concentration of one hormone led to a decrease in the concentration of another hormone. The strength of negative correlations was determined on the following scale: *r* = 0.0 to −0.2 — no correlation, *r* = −0.2 to −0.4 — weak correlation, *r* = −0.4 to −0.7 — moderate correlation, *r* = −0.7 to −0.9 — relatively strong correlation, and *r* = −0.9 to −1.0 — very strong correlation.

In group C, the correlations between the concentrations of E_2_ and P_4_ (Table 1) were absent, relatively strong and weak on successive analytical dates (these values were used as the reference in further analyses). In group ZEN5, the above correlations were evaluated as moderate on D1 and as absent on D2 and D3. In group ZEN10, a moderate negative correlation was noted on D1, and the absence of correlations was determined on D2 as well as D3. In group ZEN15, E_2_ and P_4_ concentrations were not correlated on D1, whereas moderate correlations were noted on D2 and D3.

In group C, the correlations between the concentrations of E_2_ and T (Table 2) were determined as weak, absent, and weak negative on successive analytical dates (these values were used as the reference in further analyses). In group ZEN5, the analyzed correlations were moderate on successive dates, and a moderate negative correlation was noted on D3. In group ZEN10, a moderate negative correlation was found on D1, the absence of a negative correlation was observed on D3, and the absence of a positive correlation was noted on D2 (negative value). In group ZEN15, the absence of a negative correlation was found on D1, a moderate negative correlation was observed on D3, and the absence of a positive correlation was noted on D2.

In group C, the concentrations of P_4_ and T (Table 3) were not correlated on D1, whereas moderate correlations were noted on D2 and D3 (these values were used as the reference in further analyses). In group ZEN5, a very strong correlation was observed on D1, whereas the values noted on D2 and D3 were not correlated. In group ZEN10, a relatively strong correlation was determined on D1, and the absence of correlations was noted on D2 (*r* = 0.00) and D3. In group ZEN15, no correlations were determined on D1 (*r* = 0.0), a moderate correlation was observed on D2, and a weak negative correlation was found on D3.

### 2.4. Concentrations of Zearalenone and its Metabolites in Peripheral Blood

**Figure 4:**
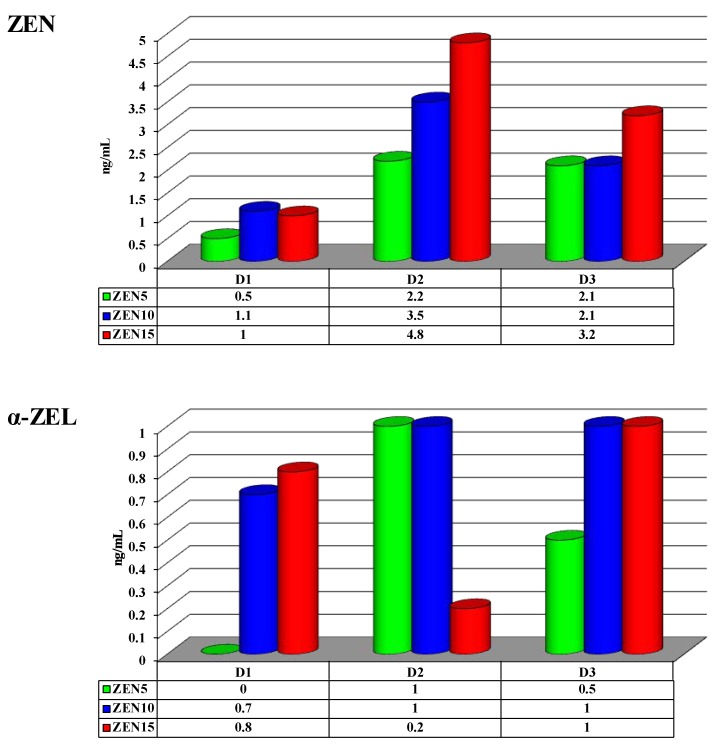

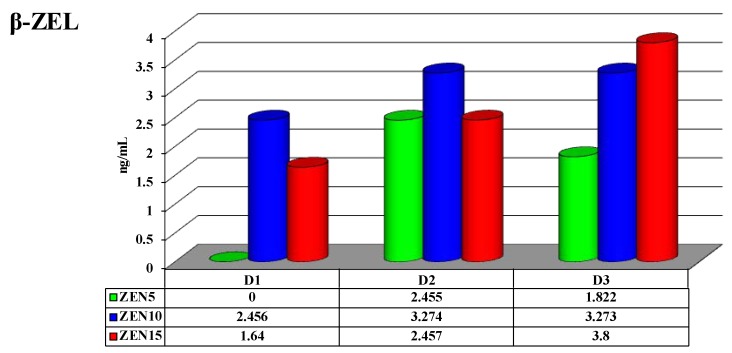
Mean ($\bar{x}$) concentrations of ZEN and its metabolites (α-ZEL and β-ZEL) (ng/mL) in the peripheral blood of pre-pubertal gilts on different analytical dates (D1 — exposure day 7; D2 — exposure day 21; D3 — exposure day 42) and in the experimental groups (group ZEN5 — 5 μg ZEN/kg BW; group ZEN10 — 10 μg ZEN/kg BW; group ZEN15 − 15 μg ZEN/kg BW). Limits of detection (LOD) > values below the limit of detection were regarded as equal to 0.

Zearalenone concentrations in the peripheral blood of pre-pubertal gilts did not differ significantly between analytical dates or groups (see Figure 4). However, considerable differences were noted in mean values. On D1, mean ZEN levels differed by 0.6 ng/mL between groups ZEN5 and ZEN10 and by 0.5 ng/mL between groups ZEN5 and ZEN15. On D2, the corresponding differences were determined at 1.3 and 2.6 ng/mL. The smallest differences between the above groups were observed on D3 at 0.0 and 1.1 ng/mL, respectively.

Similar observations were made in an analysis of ZEN metabolites, i.e., α-ZEL and β-ZEL. However, the mean levels of both metabolites differed considerably between analytical dates (see Figure 4).

On D1, the concentrations of α-ZEL differed between group ZEN5 (values below limits of detection (LOD), regarded as equal to 0) and groups ZEN10 and ZEN15 by 0.7 and 0.8 ng/mL, respectively (see Figure 4). On D2, α-ZEL levels were lowest in group ZEN15 relative to groups ZEN5 and ZEN10 (0.8 ng/mL). On D3, the differences in α-ZEL concentrations between group ZEN5 and groups ZEN10 and ZEN15 reached 0.5 ng/mL.

On D1, the concentrations of β-ZEL differed between group ZEN5 (values below LOD, regarded as equal to 0) and groups ZEN10 and ZEN15 by 2.5 and 1.6 ng/mL, respectively (see Figure 1). On D2, the corresponding differences were determined at 0.8 and 0.0 ng/mL, respectively. On D3, the respective differences were clearly pronounced at 1.5 and 2.0 ng/mL, respectively.

## 3. Discussion

The results of the present experiment validated the hypothesis that low doses of ZEN affect the concentrations of E_2_, P_4_ and T in pre-pubertal gilts on different days of exposure.

The results obtained on the first analytical date (D1) reflect the stimulatory effects of ZEN (undesirable substance) administered over a period of seven days. In the studied gilts, the effect of adaptive mechanisms, accompanied by considerable loss of energy and protein [9], was manifested on D2 [23]. The above could also be accompanied by an increase in Ca^2+^ deposition, in particular in the mitochondria [24,25], or changes in the activity of selected enzymes, such as hydroxysteroid dehydrogenases [26], which can disrupt steroidogenesis [14]. Hyperestrogenism induced by excess ZEN (that was not biotransformed or was recovered from enterohepatic circulation) probably took place on D3. “Free ZEN” can probably be utilized in specific life processes [14].

### 3.1. Estradiol

A significant or highly significant increase in E_2_ concentrations (see Figure 1) responsible for hyperestrogenism or supraphysiological hormonal levels [27] was observed in the experimental groups [2,14] relative to group C, excluding groups ZEN5 and ZEN10 on D2. The observed increase occurred as a counter-reaction to the administered doses of ZEN. On D1, E_2_ concentrations in the experimental groups relative to group C (physiological levels) were indicative of supraphysiological hormonal levels rather than hypoestrogenism (in relation to the physiological deficiency of endogenous E_2_ in group C). “Free ZEN” was captured by estrogen receptors (ERs) in the gastrointestinal tract (own study, unpublished data), and it stimulated qualitative changes (activation?) in ERs. These processes were manifested by changes in the expression of ERs, in particular ER*ß* in the descending colon, with a simultaneous quantitative increase in microbiota and an increase in genotoxicity under exposure to higher ZEN doses [7,8]. Estradiol concentrations in the peripheral blood and the metabolic profile of the studied gilts [9] indicate that “free ZEN” could: (i) induce changes during steroidogenesis (see Figure 1, group ZEN15), thus confirming that ZEN modifies the expression of enzymes such as HSD at the pre-receptor level, inversely to the applied ZEN dose [26,28], and is capable of converting T to E_2_. (ii) Increases feed intake and the accumulation of body energy reserves in gilts [9] for reproductive processes in the future [29]. The strength of linear correlations between E_2_ levels and the concentrations of P_4_ and T in the blood of pre-pubertal gilts (see Table 1 and Table 2 – moderate correlations or absence of correlations) testifies to a minor and inversely proportional (negative) effect of “free ZEN” in groups ZEN5 (MABEL dose) and ZEN10 (NOAEL dose). As a result, ZEN suppressed the concentrations of the two remaining hormones [30]. This is a significant consideration in the treatment of autoimmune disorders [23] and, more importantly, in the production of pork (sexual maturity is delayed in gilts [31,32]). In contrast, E_2_ levels in group ZEN15 (LOAEL dose) were not correlated with the concentration of P_4_ (see Table 1) on D1, whereas moderate correlations indicative of ZEN’s stimulatory effects were noted on the remaining analytical dates. In group ZEN15, the concentrations of E_2_ and T (see Table 2) were not correlated or were bound by a moderate correlation (positive correlation on D2, negative correlation on D1 and D3), which suggests that ZEN exerted weak and suppressive effects.

### 3.2. Progesterone

An analysis of P_4_ levels in the blood of pre-pubertal gilts exposed to different doses of ZEN (see Figure 2) revealed low and similar values (0.1 ng/mL) in groups C and ZEN5. Progesterone levels were determined at 0.9 ng/mL in group C only on D1. These variations are difficult to interpret [27,33]. In groups ZEN10 and ZEN15, P_4_ concentrations continued to increase throughout the experiment, which indicates that a premature increase in P_4_ levels can enhance endometrial receptivity and induce morphological changes in the reproductive system [31]. The above was accompanied by a rise in the levels of endogenous E_2_, but the correlation between E_2_ and P_4_ concentrations was negative (inversely proportional) in group ZEN10 (see Table 1). In group ZEN15, the correlation was positive. These findings suggest that the presence of MABEL and NOAEL doses of ZEN in the diet contributes to supraphysiological hormonal levels [27] and impairs P_4_ synthesis in pre-pubertal gilts long before the first estrus in pre-pubertal gilts (on D3, BW values ranged from 34 kg in group C to 41 kg in group ZEN15 [9]). In groups ZEN5 and ZEN10, P_4_ suppressed the production of antibodies, in particular asymmetrically glycosylated antibodies that are incapable of triggering immune effector mechanisms [23]. The above could be attributed to the fact that endogenous hormones and ZEN (an endocrine-disrupting chemical [6]) demonstrate hormonal activity by binding to nuclear hormone receptors (a rivalry mechanism). The consequences of the unexpected interactions [24] that take place outside the endocrine system are difficult to predict.

### 3.3. Testosterone 

Testosterone was the third analyzed hormone in the present experiment (see Figure 3). Endogenous T plays a key role in female health because it exerts direct androgenic effects or is converted to E_2_ [3]. Testosterone concentrations are characterized by significant diurnal variations [34]. They are also affected by the phase of the reproductive cycle as well as the strength and duration of exposure to stressors [35]. Testosterone regulates (i) sexual differentiation, (ii) muscle and bone mass, and (iii) erythropoietic and metabolic processes. It can exert direct and indirect biological effects through conversion to E_2_ [36]. In pre-pubertal gilts, an increase in the blood concentrations of T was directly correlated with an increase in the animals’ body weights [9]. In groups ZEN10 and ZEN15, E_2_ and T levels were bound by negative (inversely proportional) correlations (moderate correlation in group ZEN10, absence of correlations in group ZEN15) on D1. On D3, negative correlations were noted in all groups, and the highest negative values (indicative of moderate correlations) were observed in groups ZEN5 and ZEN15, whereas weak correlations were noted in the groups ZEN10 and C. These findings contradict the results reported by Kanakis et al. [34] and White et al. [35] who argued that the concentration of E_2_ in pre-pubertal gilts is influenced by the rate at which T is converted to E_2_. In pre-pubertal gilts, the levels of endogenous T do not compensate for physiological demand, and the resulting deficit is covered by “free ZEN” which changes the expression of enzymes such as HSDs [26]. However, the above does not explain the high concentration of T in all groups on D2 (see Figure 3). According to van Anders et al. [37], dominance hierarchy and animal behavior, such as maintenance of leadership and status in the herd, are affected by T, regardless of sex. This is highly probable, but in this study, ZEN was tested only in gilts of similar age and body weight that had been housed without boars. The observed differences (*P* ≤ 0.01) indicate that ZEN was a stimulating factor only in group ZEN15 (see Figure 3). Considerable variations were noted in the linear correlations between P_4_ and T levels (see Table 2). No correlations or moderate correlations between P_4_ and T levels were noted in group C. The experimental groups were characterized by extreme variations in *r* values, denoting an absence of correlations to the presence of very strong correlations, regardless of group or analytical date. These data are very difficult to interpret. It could be postulated that a LOAEL dose of ZEN increases the concentration of T on all analytical dates (see Figure 3), without inducing any correlations or promoting only weak correlations between E_2_ and T levels. However, the interactions between these hormones increase muscle mass and decrease adipose tissue mass [38]. Similar results were reported by Rykaczewska et al. [9].

### 3.4. Zearalenone (ZEN) and its Metabolites

In the current study, the absorption and biotransformation of ZEN and its metabolites in pre-pubertal gilts varied on an individual basis. Significant differences were not observed due to considerable variations in SD values (see Figure 4).

Our study demonstrated that even trace amounts of ZEN and its metabolites in peripheral blood can affect the levels of selected steroid hormones. This observation is supported by the concentrations of ZEN and its metabolites on D1 (see Figure 4) in all experimental groups. The noted levels of ZEN metabolites have not been documented in the literature. According to most studies, the biotransformation of ZEN in pigs (where 3α-HSD is more active than in other animal species) produces more α-ZEL than β-ZEL [1]. However, the noted concentrations of ZEN and its metabolites in peripheral blood could be indicative of biotransformation processes that do not induce hyperestrogenism, but alleviate the deficiency of endogenous estrogen in prepubertal gilts [27]. In group ZEN5 (MABEL dose), ZEN metabolites were not detected on D1, which can probably be attributed to the low supply of endogenous steroid hormones and the presence of exogenous ZEN. In the first case, the present results (see Figure 4) contradict other authors’ findings suggesting that the predominant ZEN metabolite in the peripheral blood of pigs is α-ZEL, rather than β-ZEL [1,14]. The above could be attributed to the higher demand for compounds with estrogenic activity, such as α-ZEL and ZEN, but not β-ZEL [39]. The noted levels of ZEN metabolites could also represent physiological values that are essential for vital life processes [2]. Our findings point to a predominance of detoxification processes in pigs that were exposed to very low doses of ZEN. It should also be noted that: (i) the seventh day of exposure to an undesirable compound such as ZEN is the final date of adaptive processes, in particular adaptive immunity [23], (ii) ZEN was utilized as a substrate (inversely proportional) regulating the expression of HSDs genes which act as molecular switches for the modulation of steroid hormones at the pre-receptor level [26,28,40], (iii) undesirable substances undergo enterohepatic circulation before they are excreted [14,41], and/or (iv) exposure to ZEN induces a specific response from intestinal microbiota [7]. The above processes, alone or in combination, can influence the peripheral blood levels of ZEN and its metabolites. Zearalenone is a promiscuous compound that can inhibit the synthesis and secretion of the follicle-stimulating hormone (FSH, [1]) via negative feedback, which decreases the production of steroid hormones [16,42].

Similar correlations between the mean concentrations of ZEN and its metabolites in peripheral blood were observed on D2 and D3 relative to D1 (see Figure 4). Zearalenone and α-ZEL levels were higher, but still low or very low. The mean concentrations of β-ZEL were similar to the values noted for ZEN on the analyzed dates. These results should be interpreted similarly to the observations made on D1. However, two differences were noted (see Figure 4). Firstly, both ZEN metabolites were present in all experimental groups. Secondly, the values of all analyzed indicators were higher, which can probably be attributed to the accumulation of ZEN and its metabolites (due to the saturation of active estrogen receptors and other factors that affect the concentrations of steroid hormones [1,43]) throughout the experiment. In the literature, the biotransformation of ZEN in peripheral blood was studied in animals exposed to higher doses of ZEN [14,44,45]. According to the hormesis principle, exposure to very low doses of ZEN [12,14,46] influences the synthesis and secretion of sex steroids [1,14,47]. The above implies that very low doses of ZEN were biotransformed in an identical manner, but the parent compound (ZEN) and its metabolites were utilized completely or to a much greater extent (which was the case in group ZEN5). The interactions between endogenous and environmental (exogenous) steroids could also be affected by other endogenous factors [15].

### 3.5. Summary

This study produced interesting observations regarding the biotransformation of ZEN in pre-pubertal gilts that were orally administered low doses of zearalenone (MABEL, NOAEL and LOAEL) over a period of 42 days. On D1, ZEN metabolites were not detected in the peripheral blood of pigs exposed to the MABEL dose. On D2 and D3 (NOAEL and LOAEL doses), the average concentration of β-ZEL in all experimental groups was three to four times higher than the concentration of α-ZEL. As a result:

- a minor increase was noted in peripheral blood levels of E2 and “free ZEN”, proportionally to the ZEN dose and analytical date;
- the concentration of E2 in peripheral blood decreased on D1 in all experimental groups and it increased on D2 and D3 in selected experimental groups (in group ZEN15 on D2, and in groups ZEN10 and ZEN15 on D3);
- testosterone levels increased significantly on all analytical dates in response to the LOAEL dose, and the concentrations of E2 and T were not correlated or were bound by weak linear correlations.

The observed endocrine effects differed in all groups due to qualitative and quantitative changes in the biotransformation of low doses of ZEN. These processes were least pronounced in the group exposed to the MABEL dose: ZEN metabolites were not detected on D1, whereas β-ZEL was the predominant metabolite on D2 and D3. The above was accompanied by an increase in the concentration of E_2_ which, together with “free ZEN”, probably suppressed P_4_ and T levels.

## 4. Materials and Methods

### 4.1. General Information

All experimental procedures involving animals were carried out in compliance with Polish regulations setting forth the terms and conditions of animal experimentation (Opinions No. 12/2016 and 45/2016/DLZ of the Local Ethics Committee for Animal Experimentation the University of Warmia and Mazury in Olsztyn, Poland in of 27 April 2016 and 30 November 2016).

### 4.2. Experimental Animals and Feed

The in vivo experiment was performed at the Department of Veterinary Prevention and Feed Hygiene of the Faculty of Veterinary Medicine at the University of Warmia and Mazury in Olsztyn on 60 clinically healthy pre-pubertal gilts with initial BW of 14.5 ± 2 kg [9]. The animals were housed in pens with free access to water. All groups of gilts received the same feed throughout the experiment. They were randomly assigned to three experimental groups (group ZEN5, group ZEN10 and group ZEN15; *n* = 15) and a control group (group C; *n* = 15 — control group) [48,49]. Group ZEN5 gilts were orally administered ZEN (Sigma-Aldrich Z2125-26MG, St. Louis, MO, USA) at 5 μg ZEN/kg BW, group ZEN10 pigs — at 10 μg ZEN/kg BW, and group ZEN15 pigs — at 15 μg ZEN/kg BW. Analytical samples of ZEN were dissolved in 96 µL of 96% ethanol (SWW 2442-90, Polskie Odczynniki SA, Poland) in weight-appropriate doses. Feed containing different doses of ZEN in an alcohol solution was placed in gel capsules. The capsules were stored at room temperature before administration to evaporate the alcohol. In the experimental groups, ZEN was administered daily in gel capsules before morning feeding. The animals were weighed at weekly intervals, and the results were used to adjust individual mycotoxin doses. Feed was the carrier, and group C pre-pubertal gilts were administered the same gel capsules, but without mycotoxins [7,8,9].

The feed administered to all experimental animals was supplied by the same producer. Friable feed was provided ad libitum twice daily, at 8:00 a.m. and 5:00 p.m., throughout the experiment. The composition of the complete diet, as declared by the manufacturer, is presented in Table 4.

The proximate chemical composition of diets fed to pigs in groups C, ZEN5, ZEN10, and ZEN15 was determined using the NIRS™ DS2500 F feed analyzer (FOSS, Hillerød, Denmark), a monochromator-based NIR reflectance and transflectance analyzer with a scanning range of 850–2500 nm.

### 4.3. Determination of Mycotoxins in Feed 

Feed was analyzed for the presence of mycotoxins and their metabolites: ZEN, α-ZEL and DON. Mycotoxin concentrations in feed were determined by separation in immunoaffinity columns (Zearala-Test^TM^ Zearalenone Testing System, G1012, VICAM, Watertown, MA, USA; DON-Test^TM^ DON Testing System, VICAM, Watertown, MA, USA) and high-performance liquid chromatography (HPLC system, Hewlett Packard type 1050 and 1100) — mass spectrometry (MS) and chromatographic column (Atlantis T3 3 µm 3.0 × 150 mm Column No. 186003723, Waters, AN Etten-Leur, Ireland). The mobile phase was a water and acetonitrile mixture with an 80:10 solvent ratio and 2 ml of CH3 COOH. The flow rate was 0.4 mL/min. The obtained values did not exceed the limits of quantitation (LoQ) of 2 ng/g for ZEN and 5 ng/g for DON. The analyzed compounds were quantified at the Department [50].

### 4.4. Blood Sampling

Blood was sampled from 5 gilts from every group on three analytical dates: exposure day 7 (D1), exposure day 21 (D2) and exposure day 42 (D3). Directly before slaughter, blood samples of 20 mL each were collected from all gilts (blood was sampled within 20 s after immobilization [51]) by jugular venipuncture into syringe containing 0.5 mL of heparin solution. Blood was centrifuged at 3000 rpm for 20 min at 4 °C. The obtained plasma samples were stored at – 18 °C until the analyses of ZEN, α-ZEL, β-ZEL, estradiol (E_2_), progesterone (P_4_) and testosterone (T) concentrations.

### 4.5. Determination of Hormone Concentrations

#### 4.5.1. Estradiol

Estradiol concentration was determined at the Institute of Animal Reproduction and Food Research of the Polish Academy of Sciences in Olsztyn, Poland. Blood plasma concentrations of E_2_ were analyzed by the radioimmunoassay (RIA) method with a commercially available kit (ESTR-US-CT, CIS BIO ASSAYS), as described previously [52,53]. All measurements were performed in duplicate for every cultured probe. The extraction yield for E_2_ was 90.67% ± 0.73%. The radioactivity of samples with J^125^ was measured with the Wallac 1470 WIZARDÆ automatic gamma scintillation counter (Perkin Elmer, Waltham, MA, USA). Radioactivity was determined within 1 min with a Geiger counter (counting efficiency, 75%). The sensitivity of the E_2_ assay was 1.36 pg/mL. The standard curve range was from 2.72 to 550 pg/mL. The intra- and inter-assay coefficients of variation were 5% and 5%, respectively.

#### 4.5.2. Progesterone and Testosterone

Progesterone and testosterone were quantified at the Analytical Laboratory of the Municipal Hospital with Polyclinic in Olsztyn, Poland, by the ECLIA electrochemiluminescence assay with the use of Elecsys Progesterone II and Elecsys Testosterone II assays and the Cobas c6000 analyzer (Hitachi, Tokyo, Japan). In the first stage, the samples were incubated with biotinylated monoclonal antibodies specific for P_4_ and T and for P_4_ and T derivatives labeled with a ruthenium complex. The extent to which the hormones were bound to antibodies was determined by their concentrations. Streptavidin-coated microspheres were added in the second stage, and the complex was bound to the solid phase during the interactions between biotin and streptavidin. The quantity of labeled P_4_ bound to the solid phase was inversely proportional to the concentration of P_4_ in the sample. The reaction mix was sucked into the measuring cell where microspheres were magnetically captured on the surface of the electrode. Unbound compounds were removed with the ProCell. Voltage was applied to the electrode, and the resulting chemiluminescence was measured with a photomultiplier. The results were read from a two-point calibration curve and a standard curve developed with a barcode verifier. The analytical range of the method was determined by the lower limit of detection and the highest point on the calibration curve at 0.03–60 ng/mL for P_4_ and 0.025–15 ng/mL for T. All determinations were performed in accordance with the manufacturer’s instructions.

#### 4.5.3. Statistical Analysis

Hormone concentrations were measured in three experimental groups and the control group on three analytical dates. The results were expressed as mean values ($\bar{x}$) and standard deviation (SD) for each sample. The following assays were performed for every hormone: (i) the differences between means were analyzed for the experimental groups and the control groups on fixed dates and (ii) the differences between means were analyzed in a fixed group on each analytical date. In both cases, the determinations were made by one-way ANOVA. If the differences between group means were statistically significant, the differences between pairs of means were determined by Tukey’s multiple comparison test. The equality of variances in the compared groups was evaluated with Levene’s test and the Brown–Forsythe test. If the equal variance hypothesis was rejected in both tests, the significance of differences was evaluated with the Kruskal–Wallis non-parametric test. In each analysis, the tested values were regarded as highly significant at *P* < 0.01 (**) and as significant at 0.01 < *P* < 0.05 (*). Linear correlations between the concentrations of steroid hormones in fixed groups were determined based on the values of the Pearson’s correlation coefficient [22]. Data were processed in Statistica v. 13 (TIBCO Software Inc., Silicon Valley, CA, USA, 2017).

### 4.6. Extraction Procedure

The presence of zearalenone, α-ZEL and β-ZEL in the blood plasma were determined with the use of immuno-affinity columns (Zearala-TestTM Zearalenone Testing System, G1012, VICAM, Watertown, MA, USA) and different protocols for each compound. All extraction procedures were performed according to the recommendations of column manufacturers. After extraction, the eluates were placed in a water bath at 50 °C, and the solvent was evaporated in a stream of nitrogen. Next, 0.5 mL of 99.8% methanol was added to dry residues to dissolve the mycotoxin.

#### 4.6.1. Quantification of ZEN and Its Metabolites

The presence of ZEN, α-ZEL and β-ZEL in the blood plasma was determined by various separation methods with the use of immuno-affinity columns (Zearala-TestTM Zearalenone Testing System, G1012, VICAM, Watertown, MA, USA) and the Agilent 1100 series liquid chromatography (LC)/mass spectrometry (MS) system. The prepared sample was estimated with the use of a chromatographic column (Atlantis T3 3 µm 3.0 × 150 mm Column No. 186003723, Waters, AN Etten-Leur, Ireland). The mobile phase consisted of 70% acetonitrile (LiChrosolvTM, No. 984 730 109, Merck-Hitachi, Mannheim, Germany), 20% methanol (LiChrosolvTM, No. 1.06 007, Merck-Hitachi, Mannheim, Germany) and 10% deionized water (Milipore Water Purification System, Millipore S.A. Molsheim-France, 2 mL of CH3 COOH). The immunoaffinity bed in the column was washed with demineralized water (Millipore Water Purification System, Millipore S.A., Molsheim, France). The flow rate was 0.4 mL/min., and the temperature of the oven column was 40 °C. The chromatographic analysis was completed in 4 min. The column was eluted with 99.8% methanol (LIChrosolvTM, No. 1.06 007, Merck-Hitachi, Mannheim, Germany) to remove the bound mycotoxin. The eluates were placed in a water bath at 50 °C, and the solvent was evaporated in a stream of nitrogen. In the next step, 0.5 mL of 99.8% methanol was added to dry residues to dissolve the mycotoxin. Mycotoxin concentrations were determined according to the external standard and were expressed in ppb (ng/mL).

Matrix-matched calibration standards were used for quantification to avoid matrix effects which can reduce sensitivity. The calibration standards were dissolved in the sample matrix prepared according to the same procedure as the remaining samples. The material used for the preparation of calibration standards was mycotoxin-free. The limits of detection (LODs) for individual mycotoxins were determined as the concentrations at which the signal-to-noise ratio decreased to 3. Alpha-ZEL and beta-ZEL were also determined. Derivative concentrations were below the LODs, and they were separated from their respective parent compounds during purification.

#### 4.6.2. Statistical Analysis

The concentrations of ZEN and its metabolites in the blood plasma of prepubertal gilts were analyzed in the control group and in three experimental groups on three analytical dates. The results were expressed as mean values ($\bar{x}$) and standard deviation (SD) for each sample. The following assays were performed for every hormone: (i) the differences between means were analyzed for the three ZEN doses (experimental groups) and the control groups on fixed dates, and (ii) the differences between means were analyzed for a fixed ZEN dose (group) on each analytical date. In both cases, the differences between means were determined by one-way ANOVA. If the differences between group means were statistically significant, the differences between pairs of means were determined with Tukey’s multiple comparison test. If all values were below the limit of detection (mean and variance equal to zero) in any group, one-way ANOVA was performed for the remaining groups (if the number of the remaining groups was higher than two), and the means of these groups were compared against zero with the use of Student’s *t*-test. The differences between groups were determined with Student’s *t*-test. In each analysis, the tested values were regarded as highly significant at *P* < 0.01 (**) and as significant at 0.01 < *P* < 0.05 (*). Data were processed in Statistica v. 13 (TIBCO Software Inc., Silicon Valley, CA, USA, 2017).

## Figures and Tables

**Table 1:** Coefficients of correlations (*r*) between the concentrations of estradiol (E_2_) and progesterone (P_4_).

Analytical Date	Group C	Group ZEN5	Group ZEN10	Group ZEN15
D1	0.105	0.445	−0.483	0.0
D2	0.767	0.0	0.0	0.594
D3	0.244	−0.231	−0.147	0.511

**Key:** Strength of linear correlations between the concentrations of E2 and P4 in the blood of pre-pubertal gilts on different analytical dates (D1 — exposure day 7; D2 — exposure day 21; D3 — exposure day 42) and in different groups where ZEN was administered once daily before the morning feeding (group C — placebo; group ZEN5 — 5 μg ZEN/kg BW; group ZEN10 — 10 μg ZEN/kg BW; group ZEN15 — 15 μg ZEN/kg BW).

**Table 2:** Coefficients of correlations (*r*) between the concentrations of E_2_ and testosterone (T).

Analytical Date	Group C	Group ZEN5	Group ZEN10	Group ZEN15
D1	0.326	0.408	−0.596	−0.015
D2	0.076	0.452	0.069	0.117
D3	−0.203	−0.492	−0.128	−0.472

**Key:** Strength of linear correlations between the concentrations of E2 and T in the blood of pre-pubertal gilts on different analytical dates (D1 — exposure day 7; D2 — exposure day 21; D3 — exposure day 42) and in different groups where ZEN was administered once daily before the morning feeding (group C — placebo; group ZEN5 — 5 μg ZEN/kg BW; group ZEN10 — 10 μg ZEN/kg BW; group ZEN15 — 15 μg ZEN/kg BW).

**Table 3:** Coefficients of correlations (*r*) between the concentrations of P_4_ and T.

Analytical Date	Group C	Group ZEN5	Group ZEN10	Group ZEN15
D1	0.220	0.998	0.730	0.0
D2	0.576	0.0	0.0	0.694
D3	0.522	0.0	0.148	−0.372

**Key:** Strength of linear correlations between the concentrations of P4 and T in the blood of pre-pubertal gilts on different analytical dates (D1 — exposure day 7; D2 — exposure day 21; D3 — exposure day 42) and in different groups where ZEN was administered once daily before the morning feeding (group C — placebo; group ZEN5 — 5 μg ZEN/kg BW; group ZEN10 — 10 μg ZEN/kg BW; group ZEN15 — 15 μg ZEN/kg BW).

**Table 4:** Declared composition of the complete diet.

Parameters	Composition Declared by The Manufacturer (%)
Soybean meal	16
Wheat	55
Barley	22
Wheat bran	4.0
Chalk	0.3
Zitrosan	0.2
Vitamin-mineral premix^1^	2.5

^1^Composition of the vitamin-mineral premix per kg: vitamin A — 500,000 IU; iron — 5000 mg; vitamin D3 — 100,000 IU; zinc — 5000 mg; vitamin E (alpha-tocopherol) — 2000 mg; manganese — 3000 mg; vitamin K — 150 mg; copper (CuSO4·5H2O) — 500 mg; vitamin B1 — 100 mg; cobalt — 20 mg; vitamin B2 — 300 mg; iodine — 40 mg; vitamin B6 — 150 mg; selenium — 15 mg; vitamin B12 — 1500 μg; L-lysine — 9.4 g; niacin — 1200 mg; DL-methionine + cystine — 3.7 g; pantothenic acid — 600 mg; L-threonine — 2.3 g; folic acid — 50 mg; tryptophan — 1.1 g; biotin — 7500 μg; phytase + choline — 10 g; ToyoCerin probiotic + calcium — 250 g; antioxidant + mineral phosphorus and released phosphorus — 60 g; magnesium — 5 g; sodium; calcium — 51 g.
